# Prenatal Diagnosis of Penoscrotal Hypospadia in Third Trimester by Two- and Three-Dimensional Ultrasonography: A Case Report

**DOI:** 10.1155/2012/142814

**Published:** 2012-12-12

**Authors:** Lívia Teresa Moreira Rios, Edward Araujo Júnior, Luciano Marcondes Machado Nardozza, Liliam Cristine Rolo, Alan Roberto Hatanaka, Antonio Fernandes Moron, Marília da Glória Martins

**Affiliations:** ^1^Mother-Child Unit, University Hospital, Federal University of Maranhão (UFMA), 65085-580 São Luiz, MA, Brazil; ^2^Department of Obstetrics, Federal University of São Paulo (UNIFESP), Rua Carlos Weber 956, Appartamento 113 Visage, Alto da Lapa, 05303-000 São Paulo, SP, Brazil

## Abstract

Hypospadia is an abnormal development of the corpus spongiosum, that involves cavernosa urethra, as a result of an inadequate fusion of the urethral folds. The incidence ranges from 0.2 to 4.1 per 1,000 live births. Among the markers of hypospadia, isolated ventral or lateral curvature of the penis associated with shortening are the most important markers and, in severe cases, can result in the classic “tulip sign.” The diagnosis of hypospadia is uncommon unless there is a routine of detailed analysis of fetal genitalia morphology. The prenatal diagnosis is of great importance for genetic counseling and allows better planning of postnatal treatment. The three-dimensional ultrasonography (3DUS) in rendering mode enables better comprehension of the pathology by parents, facilitating postnatal planning. We report a case of penoscrotal hypospadia diagnosed at 33 weeks of gestation, suspected due to the absence of testicles in the scrotum and difficulty of penis visualization. We emphasize the findings of 3DUS and its importance in the pathology compression by parents.

## 1. Introduction

Hypospadia is an abnormal development of the corpus spongiosum, that involves cavernosa urethra, as a result from an inadequate fusion of the urethral folds between the 7th and 14th weeks of pregnancy. The urethral meatus is located along the ventral surface of the penis and may terminate proximal to the glans (glandular hypospadias), at some point along the penile shaft (penile hypospadias), at the anterior margin of the scrotum (penoscrotal hypospadias), or in the perineum (perineal hypospadias) [1]. Its incidence ranges from 0.2 to 4.1 per 1,000 live births [1, 2], with a multifactorial etiology; however, a genetic predisposition has been proved [3]. The three-dimensional ultrasonography (3DUS) allows, in rendering mode, a detailed assessment of the fetal surface, allowing diagnosis often difficult using two-dimensional ultrasonography (2DUS). In relation to prenatal diagnosis of penoscrotal hypospadias, there are only three reports using 3DUS [4–6].

We report a case of penoscrotal hypospadia diagnosed at 33 weeks of gestation, suspected due to the absence of testicles in the scrotum and difficulty of penis visualization. We emphasize the findings of 3DUS and its importance in the pathology compression by parents.

## 2. Case Report

**Figure 1:**
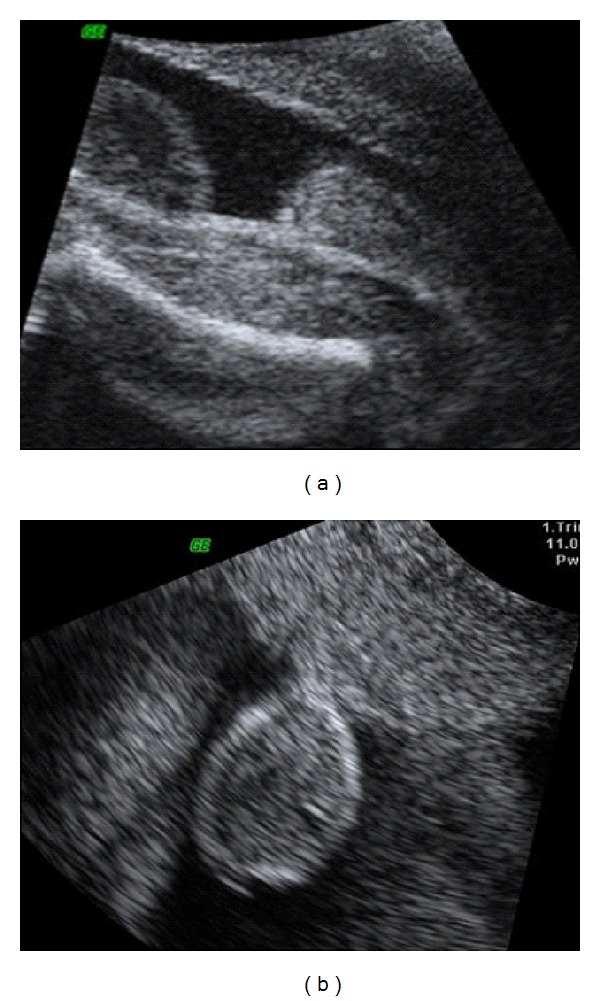
(a) and (b) Penoscrotal hypospadia assessed by two-dimensional ultrasound in the 27th week of pregnancy evaluated by abdominal approach, with clear visualization of scrotum and penis not visualized.

**Figure 2:**
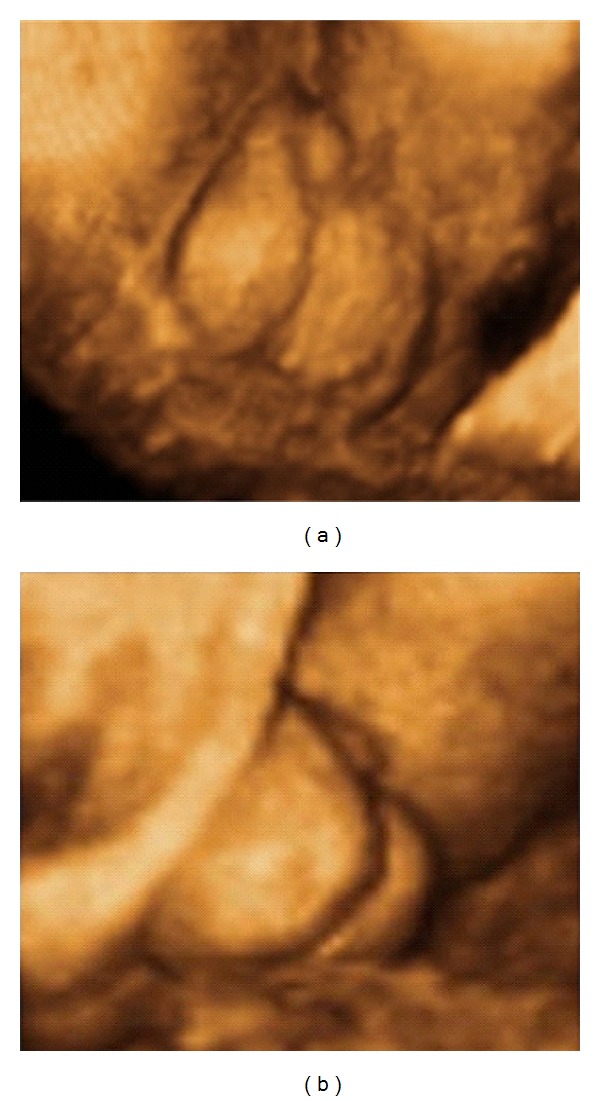
(a) and (b) Penoscrotal hypospadia assessed by three-dimensional ultrasonography in rendering mode, at 33 weeks of pregnancy, with short penis and with evidence of testicles inside a bifid scrotum.

**Figure 3:**
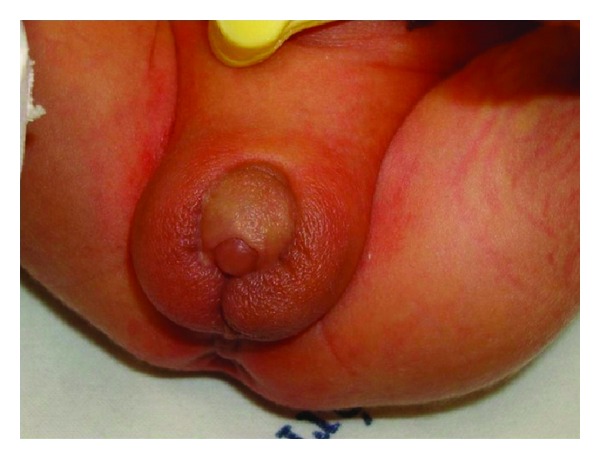
Penoscrotal hypospadia confirmed by clinical examination performed afterbirth with bifid scrotal aspect.

Primigravida of 29 years old, with 33 weeks of pregnancy, was referred to Gynecology and Obstetrics Service, University Hospital, Federal University of Maranhão (UFMA), for sonographic evaluation due to a genital abnormality detected on 2DUS performed with 27 weeks of pregnancy. 2DUS reported a male fetus with scrotum, without testicles inside and with difficult visualization of the penis (Figure 1). In order to obtain better diagnostic accuracy, we performed a 3DUS on Voluson 730 Expert (General Electric Healthcare, Zipf, Austria) using a multifrequency convex volumetric transducer (RAB 4–8L). The 3DUS in rendering mode suggested a short penis, with bifid scrotum, containing testicles inside (Figure 2). The image obtained by 3DUS had great importance for the diagnosis of penoscrotal hypospadia and provided a better understanding of the pathology by parents. The prenatal evaluation was made in the Fetal Medicine Center and the patient had birth vaginally at term. After birth, clinical examination confirmed the diagnosis of penoscrotal hypospadia (Figure 3).

## 3. Discussion

It is believed that the hypospadias results from a systemic endocrinopathy, due to inadequate response of target tissues to androgen. This lack of response is explained because of a decrease in the number of receptors and/or the inability to convert testosterone to dihidrotesterona during the critical stage of urethra morphogenesis, between the 9th and 13th weeks of gestation [7, 8]. There is a possible association of hypospadias with other malformations (neural tube, cardiac, urogenital tract, and anorectal) or a possibility to be part of a syndrome [2]. However, in most cases, hypospadia is an isolated manifestation. Therefore, only a detailed analysis of genital morphology during prenatal ultrasound allows the possibility of diagnosis [2]. The main finding of the 2DUS in cases of hypospadias is the ventral or lateral curvature of the penis, associated with its shortening. Meizner [9] described a specific signal known as a “tulip sign” present in severe hypospadias, corresponding to the presence of a short penis ventrally curved in association with penoscrotal transposition of a bifid scrotum, as described in our case. The change in morphology of distal penis, more rounded (blunt) rather than elongated (acute), results from a big and redundant prepuce only in the dorsal surface, covering the glans, that can also be observed at 2DUS [9–11].

The introduction of 3DUS allowed the evaluation of the surface structures of the fetus in rendering mode, enabling the development of a new imaging method for evaluation of hypospadias [4–6]. Fang et al. [5] described a case report of prenatal diagnosis of hypospadia in the 27th week of pregnancy, in which 3DUS rendering mode allowed a better evaluation of hypospadia. They suggest the use of 3DUS in cases of reduced penis length or altered form analyzed with 2DUS. Wang et al. [6] described two cases of prenatal diagnosis of penoscrotal hypospadias using 3DUS, with 27 and 32 weeks. These authors reported that 3DUS provides realistic images that can be shared by other professionals such as pediatric surgeons.

In rendering mode, it is possible to identify more details of abnormal genitalia, often difficult to observe only with 2DUS. Thus, the broader and accurate screening of some superficial malformations rarely detected on routine scan, such as hypospadias, becomes feasible even during the prenatal period, allowing a complete and detailed evaluation of the fetus. It also allows parents to better understand fetal pathology, providing better counseling.
